# Investigating the Role of Cannabinoid Type 1 Receptors in Vascular Function and Remodeling in a Hypercholesterolemic Mouse Model with Low-Density Lipoprotein–Cannabinoid Type 1 Receptor Double Knockout Animals

**DOI:** 10.3390/ijms25179537

**Published:** 2024-09-02

**Authors:** Zsolt Vass, Kinga Shenker-Horváth, Bálint Bányai, Kinga Nóra Vető, Viktória Török, Janka Borbála Gém, György L. Nádasy, Kinga Bernadett Kovács, Eszter Mária Horváth, Zoltán Jakus, László Hunyady, Mária Szekeres, Gabriella Dörnyei

**Affiliations:** 1 Department of Morphology and Physiology, Faculty of Health Sciences, Semmelweis University, 17 Vas Street, 1088 Budapest, Hungary; vass.zsolt@semmelweis.hu (Z.V.); shenker-horvath.kinga@tf.hu (K.S.-H.); kinga011098@gmail.com (K.N.V.); viktoria0529@gmail.com (V.T.); dornyei.gabriella@semmelweis.hu (G.D.); 2 Center for Sports Nutrition Science, Hungarian University of Sports Science, 42-48 Alkotás Street, 1123 Budapest, Hungary; 3 Department of Physiology, Faculty of Medicine, Semmelweis University, 37-47 Tűzoltó Street, 1094 Budapest, Hungary; banyai.balint.peter@semmelweis.hu (B.B.); gem.janka@phd.semmelweis.hu (J.B.G.); nadasy.gyorgy.laszlo@semmelweis.hu (G.L.N.); kovacs.kinga@phd.semmelweis.hu (K.B.K.); horvath.eszter@med.semmelweis-univ.hu (E.M.H.); jakus.zoltan@semmelweis.hu (Z.J.); hunyady.laszlo@med.semmelweis-univ.hu (L.H.); 4 Institute of Molecular Life Sciences, HUN-REN Research Centre for Natural Sciences, 2 Magyar Tudósok Körútja, 1117 Budapest, Hungary

**Keywords:** cholesterol, hypercholesterolemia, atherosclerosis, CB_1_ receptor, LDL receptor, endocannabinoid, vascular remodeling, high-fat diet

## Abstract

Hypercholesterolemia forms the background of several cardiovascular pathologies. LDL receptor-knockout (LDLR-KO) mice kept on a high-fat diet (HFD) develop high cholesterol levels and atherosclerosis (AS). Cannabinoid type 1 receptors (CB_1_Rs) induce vasodilation, although their role in cardiovascular pathologies is still controversial. We aimed to reveal the effects of CB_1_Rs on vascular function and remodeling in hypercholesterolemic AS-prone LDLR-KO mice. Experiments were performed on a newly established LDLR and CB_1_R double-knockout (KO) mouse model, in which KO and wild-type (WT) mice were kept on an HFD or a control diet (CD) for 5 months. The vascular functions of abdominal aorta rings were tested with wire myography. The vasorelaxation effects of acetylcholine (Ach, 1 nM–1 µM) were obtained after phenylephrine precontraction, which was repeated with inhibitors of nitric oxide synthase (NOS) and cyclooxygenase (COX), Nω-nitro-L-arginine (LNA), and indomethacin (INDO), respectively. Blood pressure was measured with the tail-cuff method. Immunostaining of endothelial NOS (eNOS) was carried out. An HFD significantly elevated the cholesterol levels in the LDLR-KO mice more than in the corresponding WT mice (mean values: 1039 ± 162 mg/dL vs. 91 ± 18 mg/dL), and they were not influenced by the presence of the CB_1_R gene. However, with the defect of the CB_1_R gene, damage to the Ach relaxation ability was moderated. The blood pressure was higher in the LDLR-KO mice compared to their WT counterparts (systolic/diastolic values: 110/84 ± 5.8/6.8 vs. 102/80 ± 3.3/2.5 mmHg), which was significantly elevated with an HFD (118/96 ± 1.9/2 vs. 100/77 ± 3.4/3.1 mmHg, *p* < 0.05) but attenuated in the CB_1_R-KO HFD mice. The expression of eNOS was depressed in the HFD WT mice compared to those on the CD, but it was augmented if CB_1_R was knocked out. This newly established double-knockout mouse model provides a tool for studying the involvement of CB_1_Rs in the development of hypercholesterolemia and atherosclerosis. Our results indicate that knocking out the CB_1_R gene significantly attenuates vascular damage in hypercholesterolemic mice.

## 1. Introduction

Hypercholesterolemia (HC) is a leading cardiovascular risk factor (CVRF) that predisposes individuals to cardiovascular diseases, such as atherosclerosis (AS) and hypertension (HT), which may alter vascular functions by causing endothelial dysfunction [1,2,3,4,5,6]. During the pathogenesis of cardiovascular diseases (CVDs), long-term exposure to low-density lipoprotein (LDL) and other CVRFs increases the prevalence of subclinical AS and is associated with the risk of cardiovascular events occurring later, such as lethal ischemic heart diseases or stroke, which are mainly caused by chronic arterial inflammation and slow lipid build-up in the walls of larger vessels and the development of AS [7,8]. LDL receptor signaling plays a major role in the development of AS [9]. Cardiovascular diseases and ischemic heart diseases are growing in number globally, mostly due to the growth of the older population [4].

To examine CVRFs and AS, genetically modified experimental models of mice are widely accepted [10,11,12]. Such models include the Apo-E-knockout mouse strain, LDL receptor-knockout (LDLR-KO) mice, or Apo-E–LDLR double-KO mice. Wild types of the C57BL/6 mouse strain are naturally resistant to AS and are widely used as controls. Apo-E-KO mice develop pathologic lipid profiles, decreased high-density lipoprotein (HDL) levels, and elevated very-low-density lipoprotein (VLDL) levels, thus developing AS even if kept on a regular chow diet. LDLR-KO mice kept on a high-fat diet (HFD) are relevant mouse models for the human familial hypercholesterolemia and are commonly used in animal experiments investigating hyperlipidemia and AS; these mice develop cholesterol levels as high as 800–1000 mg/dL when kept on a long-term HFD [7,11,12,13,14]. Hypercholesterolemic LDLR-KO mice kept on an HFD develop atherosclerotic plaques in their thoracic aorta with altered endothelium-mediated vasodilation in their arteries [15]. Other AS-prone animal models are also used, such as Apo-E3-Leiden and PCSK9-AAV mice and Apo-E−/−Fbn1C1039G+/− mice, which can develop intra-plaque microvessels, hemorrhages, spontaneous atherosclerotic plaque ruptures, and myocardial infarction and experience sudden cardiac death [11,12].

The endocannabinoid system (ECS) has a wide range of effects on physiological functions, influencing the neuroendocrine and cardiovascular systems, the immune and digestive systems, reproductive functions, the cell cycle, body temperature, and bone formation, as well as other aspects of human physiology, including appetite control and neurobehavioral and analgesic pathways [2,16,17]. The ECS activates type 1 cannabinoid receptors (CB_1_Rs) and type 2 cannabinoid receptors (CB_2_Rs), members of the G-protein-coupled receptor (GPCR) family, which are the main targets of active compounds from *Cannabis sativa* such as tetrahydrocannabinol (THC). The endogenous agonists of these receptors are endocannabinoids (eCBs), such as anandamide (AEA) and 2-arachidonoylglycerol (2-AG), which are synthesized by the enzymes diacylglycerol (DAG) lipase, NAPE-PLD, and degraded by the monoacylglycerol (MAG) lipase, fatty acid amide hydrolase (FAAH) [2,17,18,19,20,21,22,23,24,25]. AEA and 2-AG bind to both cannabinoid receptors: AEA has a higher affinity, whereas 2-AG has a higher efficacy on them [26]. Altogether, 13 compounds of eCBs have been identified [27]. Cannabinoid receptors are also activated by several synthetic agonists, such as WIN55,212-2 or HU-120, and synthetic antagonists such as AM251, O2050, or rimonabant also exist [2,19,24,28,29]. The physiological roles of the ECS are diverse, as it participates in several regulatory mechanisms, such as synaptic neurotransmission, cardiovascular effects, metabolism, appetite control, pain perception, overall well-being, and memory functions. Several metabolic control processes are modified by the ECS; CB_1_R-dependent signaling increases appetite and promotes weight gain. The ECS also influences the endocrine system, such as the hypothalamus–pituitary axis, among others, modulating the release of gonadotropin-releasing hormone (GnRH). These interactions highlight the complex nature of the ECS in controlling various physiological processes [2,17,19,23,24,30,31,32]. Both CBR agonists and CBR antagonists can be used for clinical therapy purposes. CB_1_R antagonists have been tested for the treatment of obesity, although the CB_1_R antagonist rimonabant had to be withdrawn due to its unwanted side effects [2,33].

The ECS has significant effects on cardiovascular functions. Previous studies have indicated negative cardiac inotropic, vasodilator, and hypotensive effects via CB_1_R signaling mechanisms [2,24,28,29,32]. The GPCR signaling-induced release of eCBs by several agonists has been found to attenuate vasoconstriction effects through the coactivation of CB_1_Rs [24,29,30,34,35]. Our previous studies indicated that the CB_1_R agonist WIN 55,212-2 induced vasodilatory effects, which were missing in CB_1_R-KO mice [24,29,36]. In the absence of CB_1_Rs in female mice (life-long effects), however, enhanced vasodilatory abilities were found, mediated by enhanced endothelial nitric oxide (NO) and altered endogenous prostanoid (PG) release. Endogenous CBs via the activation of CB_1_Rs were identified as significant contributors to the structural remodeling of the vascular wall in a recent paper from our laboratory [36]. ECS signaling was shown to affect the development of AS and plaque stability via multiple mechanisms, such as vascular inflammation, cholesterol metabolism, and leukocyte recruitment [37].

To reveal the potential roles and mechanisms of CB_1_Rs in vascular wall remodeling in hypercholesterolemic AS-prone LDLR-KO mice, we have developed a double-knockout mouse model, specifically an LDLR-KO and CB_1_R-KO mouse model, through targeted breeding processes. By keeping these animals on an HFD, we were able to investigate the effects of the existence of the CB_1_R on the functional and structural remodeling of the aortic wall in hypercholesterolemic AS-prone mice. By establishing this LDLR–CB_1_R double-knockout mouse strain, we have developed a mouse model suitable for studying the involvement of CB_1_Rs in the development of hypercholesterolemia and atherosclerosis.

## 2. Results

### 2.1. Body Weight and Heart Weight Values

**Figure 1:**
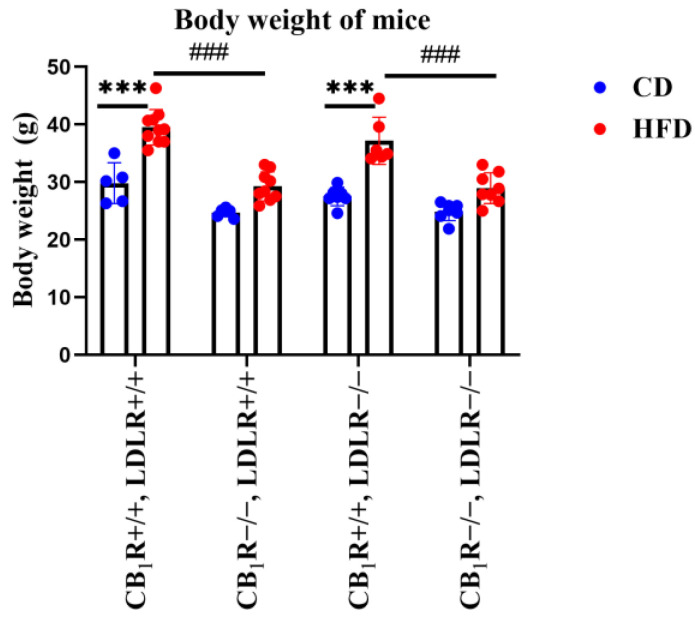
Body weight values of mice with wild-type/knocked-out CB_1_R and LDLR kept on a control diet or a high-fat diet (one-way ANOVA with pairwise comparisons and Bonferroni post hoc test) (***, *p* < 0.001; ###, *p* < 0.001; *n* = 5–10). Mean ± SEM values are indicated here, with dots showing individual data points. Abbreviations: CD, control diet; HFD, high-fat diet; CB_1_R+/+, CB_1_R wild type; CB_1_R−/−, CB_1_R knockout; LDLR+/+, low-density lipoprotein receptor wild type; LDLR−/−, low-density lipoprotein receptor knockout.

The high-fat diet significantly increased the body weight of the mice (Figure 1), an effect that was less pronounced in the CB_1_R-KO mice (*p* < 0.001 HFD vs. CD; *p* < 0.001 CB_1_R+/+ vs. CB_1_R−/−). Heart weight values slightly increased under the HFD, which was not a significant effect. A significantly elevated heart weight could be seen in the CB_1_R+/+, LDLR+/+, HFD group compared to the CB_1_R−/−, LDLR+/+, CD group (*p* = 0.007; Supplementary Figure S1).

### 2.2. Cholesterol Level Measurements

**Figure 2:**
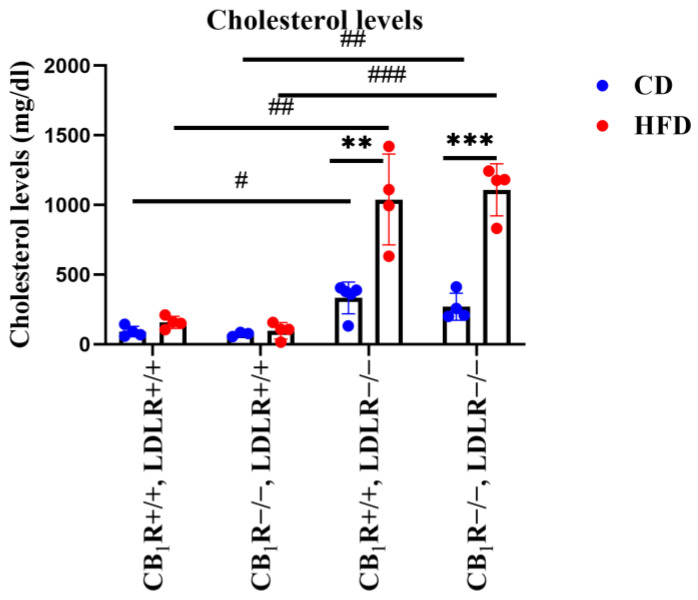
Cholesterol levels of mice with wild-type/knocked-out CB_1_R and LDLR kept on a control diet or a high-fat diet (one-way ANOVA with Holm–Sidak comparisons and two-way ANOVA with Bonferroni post hoc test) (**, *p* = 0.006 and ***, *p* < 0.001 between CD and HFD groups) (#, *p* = 0.013; ##, *p* = 0.002; and ###, *p* < 0.001 between LDLR+/+ and LDLR−/− groups) (*n* = 3–5). Mean ± SEM values are indicated here, with dots showing individual data points. Abbreviations: CD, control diet; HFD, high-fat diet; CB_1_R, cannabinoid type 1 receptor; CB_1_R+/+, endocannabinoid type 1 receptor wild type; CB_1_R−/−, endocannabinoid type 1 knockout; LDLR, low-density lipoprotein receptor; LDLR+/+, low-density lipoprotein receptor wild type; LDLR−/−, low-density lipoprotein receptor knockout.

We investigated the effects of the HFD on plasma cholesterol levels in our animal model. We found that plasma cholesterol concentrations did not differ significantly in the LDLR+/+ groups. In LDLR+/+ animals, the HFD elevated plasma cholesterol levels compared to CD-fed animals, but this effect did not reach the level of statistical significance (Figure 2). However, in LDLR−/− groups, the HFD induced a pronounced elevation of the plasma cholesterol concentrations, far into the level of pathological hypercholesterolemia compared to the CD (*p* = 0.001 between CB_1_R−/−, LDLR−/−, HFD mice and CB_1_R−/−, LDLR−/−, CD mice; *p* = 0.006 between CB_1_R+/+, LDLR−/−, HFD mice and CB_1_R+/+, LDLR−/−, CD mice). Further, LDLR−/− animals, even if kept on the CD, presented an increased cholesterol concentration compared to LDLR+/+ animals on the control diet, independently of the presence of the CB_1_ receptor (*p* = 0.013 between CB_1_R+/+, LDLR+/+, CD mice and CB_1_R+/+, LDLR−/−, CD mice; *p* = 0.002 between CB_1_R−/−, LDLR+/+, CD mice and CB_1_R−/−, LDLR−/−, CD mice). There were no significant differences in serum cholesterol levels between the CB_1_R-wild-type and CB_1_R-KO groups (Figure 2).

### 2.3. Blood Pressure Measurements

**Figure 3:**
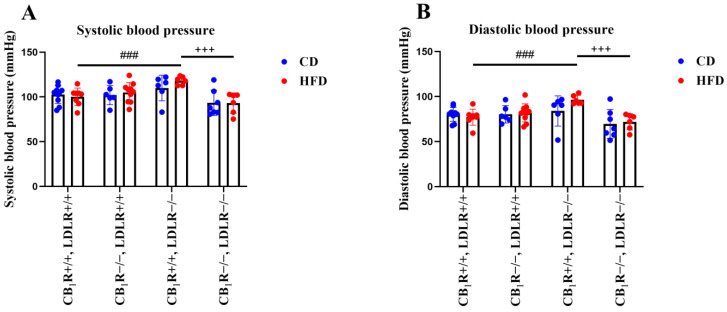
Systolic and diastolic blood pressures of mice with wild-type/knocked-out CB_1_R and LDLR kept on a control diet or a high-fat diet. Panel (**A**): Systolic blood pressure of mice with wild-type/knocked-out CB_1_R and LDLR kept on a control diet or a high-fat diet (*n* = 5–10). Panel (**B**): Diastolic blood pressure of mice with wild-type/knocked-out CB_1_R and LDLR kept on a control diet or a high-fat diet (*n* = 5–10). +++, *p* < 0.001 between CB_1_R+/+ and CB_1_R−/−; ###, *p* < 0.001 between LDLR+/+ and LDLR−/− (one-way ANOVA with Holm–Sidak pairwise comparisons, *n* = 5–10). Mean ± SEM values are indicated here, with dots showing individual data points. Abbreviations: CD, control diet; HFD, high-fat diet; CB_1_R+/+, endocannabinoid type 1 receptor wild type; CB_1_R−/−, endocannabinoid type 1 knockout; LDLR+/+, low-density lipoprotein receptor wild type; LDLR−/−, low-density lipoprotein receptor knockout.

During Euthasol anesthesia, there were elevated systolic and diastolic BP values in the LDLR−/−, CB_1_R+/+, HFD animals compared to the LDLR+/+, CB_1_R+/+, HFD animals (*p* < 0.001, Figure 3A,B); such elevation was not observed in the CB_1_R−/−, LDLR−/−, HFD group (resulting in a statistical difference, *p* < 0.001, between the CB_1_R +/+, LDLR−/− and CB_1_R−/−, LDLR−/−, HFD groups). There was no statistical difference in the systolic and diastolic blood pressures between CD and HFD groups in the LDLR−/− and CB_1_R+/+ genotype (Figure 3A,B).

### 2.4. Endothelium-Dependent Vasodilation of Abdominal Aortic Segments

**Figure 4:**
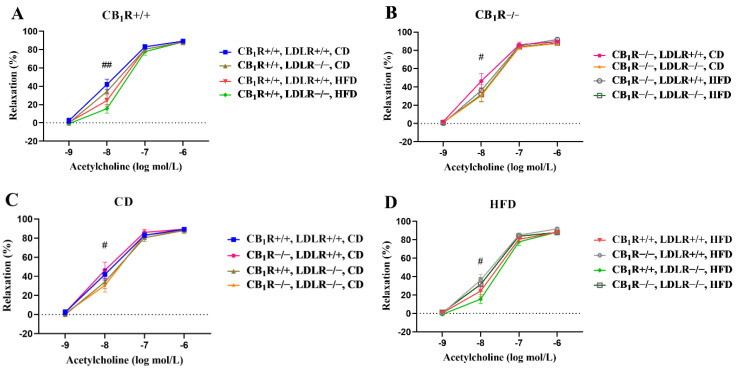
Acetylcholine-induced endothelium-dependent vasodilation in aortic segments of mice with wild-type/knocked-out CB_1_R and LDLR kept on a control or a high-fat diet. Panel (**A**): Dose–response relaxation curves in relation to Ach in CB_1_R-wild-type groups with different LDLR genotypes and diets (##, *p* = 0.008; one-way ANOVA with Bonferroni post hoc test; *n* = 5–10). Panel (**B**): Dose–response relaxation curves in relation to Ach in CB_1_R-knockout groups with different LDLR genotypes and diets (#, *p* = 0.041; two-way ANOVA with Holm–Sidak post hoc test; *n* = 6–9). Panel (**C**): Dose–response relaxation curves in relation to Ach in control-diet groups with different LDLR and CB_1_R genotypes (#, *p* = 0.047; two-way ANOVA with Holm–Sidak post hoc test; *n* = 5–7). Panel (**D**): Dose–response relaxation curves in relation to Ach in HFD groups (*n* = 5–10) with different LDLR and CB_1_R genotypes (#, *p* = 0.043; one-way ANOVA with Bonferroni post hoc test). Data are shown as mean ± SEM values. Relaxation data were calculated as percent values of the precontraction level. Abbreviations: CD, control diet; HFD, high-fat diet; CB_1_R+/+, endocannabinoid type 1 receptor wild type; CB_1_R−/−, endocannabinoid type 1 receptor knockout; LDLR+/+, low-density lipoprotein receptor wild type; LDLR−/−, low-density lipoprotein receptor knockout.

Endothelium-dependent vasodilator Ach induced dose-dependent vasodilation in all groups (Figure 4A–D). There was an overall statistical difference between the CD and HFD groups according to the two-way ANOVA (*p* = 0.026). Ach-induced relaxation was the best in the CB_1_R+/+, LDLR+/+ and CB_1_R+/+, LDLR−/−, CD groups, which was attenuated due to the HFD in the groups of the same genotype (significant between CB_1_R+/+, LDLR+/+, CD and CB_1_R+/+, LDLR−/−, HFD, *p* = 0.008). The difference between the CB_1_R+/+, LDLR−/−, HFD and CD groups did not reach the level of statistical significance (*p* = 0.064; Figure 4A). There was, however, a statistical difference between the animals kept on high-fat and low-fat diets when the CB_1_R was missing, with the CB_1_R−/−, LDLR+/+, CD group showing the best relaxation (*p* = 0.041; Figure 4B).

The Ach-induced relaxation was better in the LDLR+/+, CD groups compared to the LDLR−/−, CD groups (*p* = 0.047). Relaxation was improved in the CB_1_R−/−, CD groups vs. the CB_1_R+/+, CD groups. This relaxation was improved by knocking out the CB_1_ receptor (*p* = 0.016, two-way ANOVA with Holm–Sidak test; Figure 4C).

There was no difference in Ach-induced relaxation regarding the presence of the LDL receptor (LDLR+/+ vs. LDLR−/− in HFD groups); also, there was no such statistical difference in the case of CB_1_Rs in the HFD groups.

The worst relaxation for Ach (10^−8^ mol/L) was observed in CB_1_R+/+, HFD groups. The relaxation was significantly improved in the CB_1_R−/−, LDLR+/+, HFD group compared to the CB_1_R+/+, LDLR−/−, HFD group (*p* = 0.043; Figure 4D).

There was also a statistical difference between the CB_1_R+/+, LDLR−/−, HFD and CB_1_R−/−, LDLR+/+, CD groups, in which the HFD-fed mice showed the worst relaxation to Ach at the dosage of 10^−8^ mol/L (*p* = 0.015, one-way ANOVA, Bonferroni post hoc test).

Ach-induced relaxation responses were also analyzed with the curve-fitting method. When comparing the maximum effects (Emax) and the effective concentration at 50% of the maximum response (EC50) on the Ach-induced dose–response curves, differences in EC50 values showed significance between the CB_1_R+/+, LDLR−/−, CD and CB_1_R+/+, LDLR−/−, HFD groups (*p* = 0.043), indicating that HFD treatment ameliorated Ach-induced vasodilation by shifting the EC50 value upward (from 13.4 ± 2.9 to 26.4 ± 5.3 nmol/L; Supplementary Table S1). Regarding the role of CB_1_Rs, EC50 was decreased significantly in the CB_1_R−/−, LDLR−/−, HFD group compared to the CB_1_R+/+, LDLR−/−, HFD animals (from 26.4 ± 5.3 to 14.5 ± 3.0 nmol/L, *p* < 0.05) indicating an improvement in vasodilation in the absence of CB_1_Rs (Supplementary Table S1).

### 2.5. Effects of Specific Inhibitors on Acetylcholine-Induced Vasodilatory Responses

**Figure 5:**
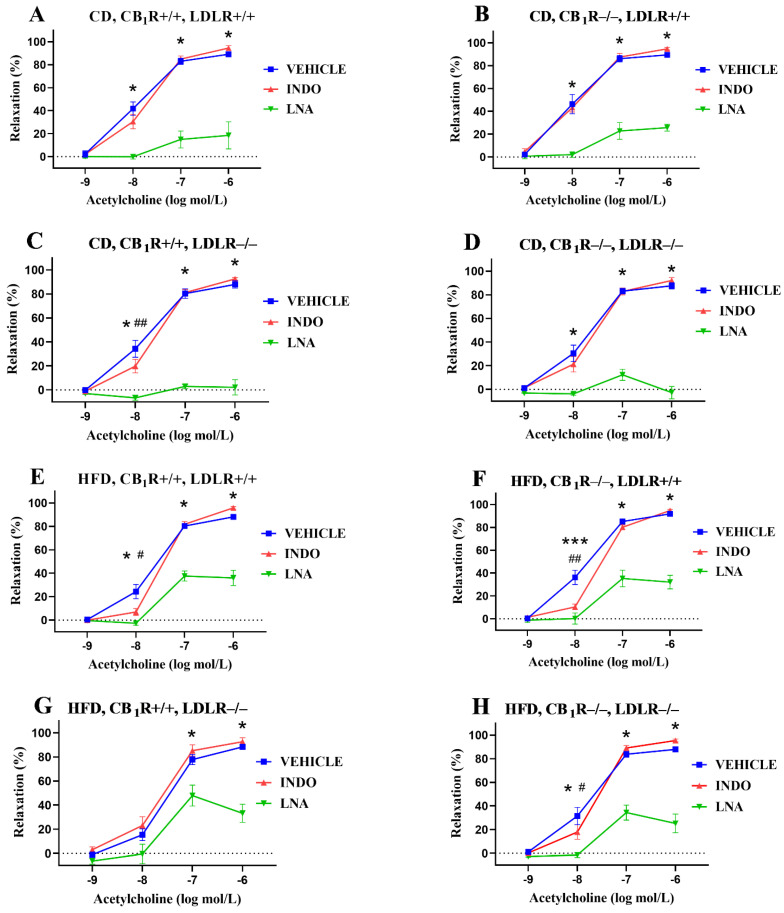
Effects of specific inhibitors (Nω-nitro-L-arginine and indomethacin, inhibitors of NOS and COX, respectively) on acetylcholine-induced relaxation responses in aortas of mice with wild-type/knocked-out CB_1_R and LDLR kept on a control diet or a high-fat diet. Panel (**A**): Effects of inhibitors on acetylcholine-induced vasodilation in CB_1_R+/+, LDLR+/+, control-diet mice (*n* = 5–6). Panel (**B**): Effects of inhibitors on acetylcholine-induced vasodilation in the CB_1_R−/−, LDLR+/+, control-diet group (*n* = 6). Panel (**C**): Effects of inhibitors on acetylcholine-induced vasodilation in CB_1_R+/+, LDLR−/−, control-diet group (*n* = 7). Panel (**D**): Effects of inhibitors on acetylcholine-induced vasodilation in the CB_1_R−/−, LDLR−/−, control-diet group (*n* = 6). Panel (**E**): Effects of inhibitors on acetylcholine-induced vasodilation in CB_1_R+/+, LDLR+/+, high-fat-diet mice (*n* = 9–10). Panel (**F**): Effects of inhibitors on acetylcholine-induced vasodilation in CB_1_R−/−, LDLR+/+, high-fat-diet mice (*n* = 9). Panel (**G**): Effects of inhibitors on acetylcholine-induced vasodilation in CB_1_R+/+, LDLR−/−, high-fat-diet mice (*n* = 5). Panel (**H**): Effects of inhibitors on acetylcholine-induced vasodilation in the CB_1_R−/−, LDLR−/−, high-fat-diet group (*n* = 7). Data are shown as mean ± SEM values. *p* < 0.05 values were considered significant. *, *p* < 0.05 and ***, *p* < 0.001 between vehicle- and LNA-treated groups; #, *p* < 0.05 and ##, *p* < 0.01 between vehicle- and INDO-treated groups. Statistics were calculated via a one-way ANOVA with Holm–Sidak comparisons or a two-way ANOVA with the Bonferroni post hoc test. Ranking was made with Kruskal–Wallis and Dunn’s tests. Abbreviations: CD, control diet; HFD, high-fat diet; INDO, indomethacin; LNA, Nω-nitro-L-arginine; CB_1_R+/+, cannabinoid type 1 receptor wild type; CB_1_R−/−, cannabinoid type 1 receptor knockout; LDLR+/+, LDL receptor wild type; LDLR−/−, LDL receptor knockout. Relaxation data were calculated as percent values of the precontraction level.

The inhibition of nitric oxide synthase with LNA significantly decreased the Ach-induced relaxation in all groups in concentrations of 10^−8^–10^−6^ mol/L (except in the CB_1_R+/+, LDLR−/−, HFD group at 10^−8^ mol/L, where a non-significant reduction in relaxation could be seen; Figure 5A–H). The inhibition of cyclooxygenase with INDO slightly modulated Ach-induced relaxation, which was significantly decreased at 10^−8^ mol/L in both the CB_1_R+/+, LDLR−/−, CD and CB_1_R+/+, LDLR+/+, HFD groups, as well as in the CB_1_R−/−, HFD groups (Figure 5C,E,F,H).

### 2.6. Comparison of the Effects of NOS Inhibitor LNA on Acetylcholine-Induced Vasodilatory Responses

**Figure 6:**
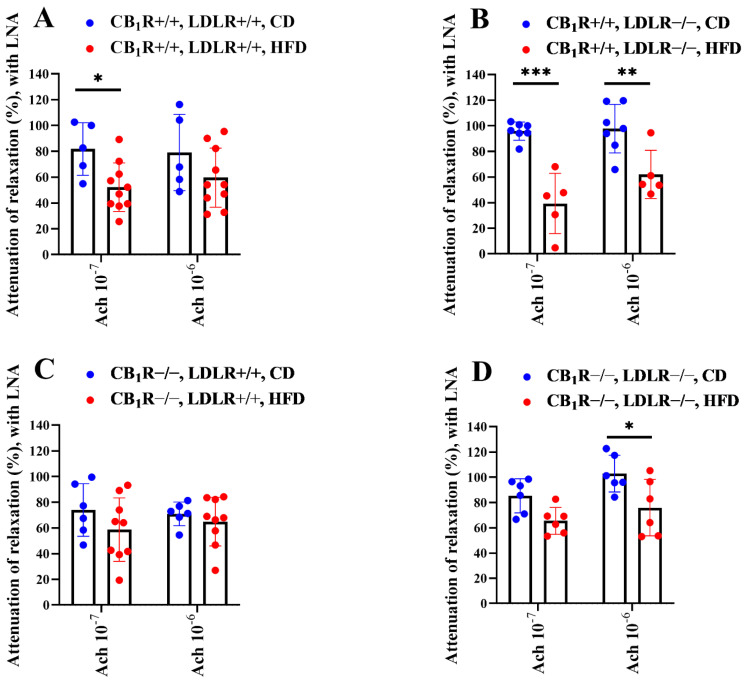
Effects of the specific inhibitor Nω-nitro-L-arginine on Ach-induced relaxation in aortas of mice with wild-type/knocked-out CB_1_R and LDLR kept on a control or a high-fat diet, normalized to control values. Attenuation of Ach-induced relaxation with LNA is shown in percent values. Panel (**A**): Attenuation of Ach-induced relaxation with LNA in CB_1_R+/+, LDLR+/+, CD and HFD groups, *n* = 5–10. Panel (**B**): Attenuation of Ach-induced relaxation with LNA in CB_1_R+/+, LDLR−/−, CD and HFD groups, *n* = 5–7. Panel (**C**): Attenuation of Ach-induced relaxation with LNA in CB_1_R−/−, LDLR+/+, CD and HFD groups, *n* = 6–9. Panel (**D**): Attenuation of Ach-induced relaxation with LNA in CB_1_R−/−, LDLR−/−, CD and HFD groups, *n* = 6–7. *p*-values < 0.05 were considered significant. *, *p* < 0.05; **, *p* < 0.01; ***, *p* < 0.001 between CD and HFD groups in the same genotype (one-way ANOVA with Bonferroni post hoc test). Mean ± SEM values are indicated here, with dots showing individual data points. Abbreviations: CD, control diet; HFD, high-fat diet; LNA, Nω-nitro-L-arginine; CB_1_R+/+, cannabinoid type 1 receptor wild type; CB_1_R−/−, cannabinoid type 1 receptor knockout; LDLR+/+, LDL receptor wild type; LDLR−/−, LDL receptor knockout. Relaxation data were calculated as percent values of the precontraction level.

NOS inhibition decreased Ach-induced relaxation in all groups (Figure 5A–H), which was greater in all CD groups compared to HFD groups. This difference was significant in pairwise comparisons in the case of CB_1_R+/+, LDLR+/+ and CB_1_R+/+, LDLR−/− genotypes, as well as in CB_1_R−/−, LDLR−/− genotypes (Figure 6A–D). In CB_1_R-KO groups, the differences in LNA-induced attenuation of Ach relaxation were less pronounced between CD and HFD groups (significant only between groups in double-knockout mice at an Ach concentration of 10^−6^ mol/L; Figure 6C,D) compared to the CB_1_R-wild-type groups (Figure 6A,B).

### 2.7. Immunohistochemistry Results for Endothelial NOS

**Figure 7:**
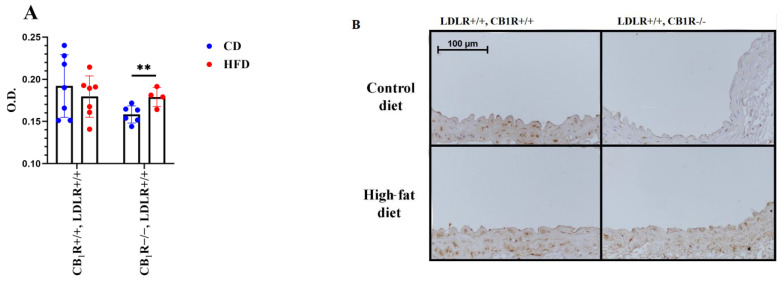
Expression of endothelial nitric oxide synthase (eNOS) in abdominal aortas of LDLR+/+ mice with wild-type or knocked-out CB_1_R kept on a control diet or a high-fat diet, *n* = 4–7. **, *p* = 0.016 between control-diet (CD) and high-fat-diet (HFD) groups in the same genotype. Mean ± SEM values are indicated here, with dots showing individual data points. Abbreviations: CD, control diet; HFD, high-fat diet; OD, optical density; CB_1_R+/+, cannabinoid type 1 receptor wild type; CB_1_R−/−, cannabinoid type 1 receptor knockout; LDLR+/+, LDL receptor wild type. Panel (**A**): Optical density (OD) levels of eNOS expression. Panel (**B**): Representative photos indicating eNOS expression chosen form 4–7 slides of each group.

The expression of endothelial NOS was measured in LDLR+/+ groups to study the effects of diet and CB_1_ receptor genotype on this expression. Samples were taken from the upper level of the abdominal aorta between the diaphragm and the renal arteries. We found that in CB_1_R+/+, HFD groups, the eNOS expression was slightly decreased, which was reversed in CB_1_R-KO groups, with this difference being statistically significant. Thus, the absence of CB_1_ receptors resulted in higher eNOS abundance in HFD groups compared with CD animals (Figure 7A,B). This altered expression can partially explain our results on myography, which indicated that during the inhibition of NO production with LNA in CB_1_R+/+ animals, Ach-induced vasodilation was attenuated by HFD (Figure 6A), the lesser sensitivity to HFD of CB_1_R−/− animals (Figure 6C) can be the result of an elevated eNOS expression (Figure 7). 

## 3. Discussion

The main findings of our study indicate that HFD-induced vascular functional damage is attenuated in the absence of CB_1_Rs.

We have demonstrated the effectivity of an HFD treatment protocol in experimental animals, as it increased the body weights of mice in our study, while also increasing cholesterol levels in LDLR-KO mice. In the LDLR-KO, HFD-fed mice, the cholesterol levels reached values over 1000 mg/dL, which represents a serious case of hypercholesterolemia. In CB_1_R-KO mice, body weights were significantly lower compared to wild-type mice, as has been shown before [2], and this difference remained in HFD-fed groups. A lack of LDL receptors substantially elevated the plasma cholesterol level, and the absence of CB_1_Rs did not prevent this parameter from rising in HFD-fed animals. So, based on our observations, we can practically exclude the possibility that the observed vascular defense actions of knocking out the CB_1_R could be connected to plasma cholesterol reduction. Considering cardiovascular parameters, the HFD increased systolic and diastolic BP values in LDLR-knockout CB_1_R+/+ mice compared to LDLR+/+, CB_1_R+/+ mice, which was attenuated in CB_1_R-KO mice. The HFD also reduced Ach-induced endothelium-dependent relaxation compared to CD groups, which was more prominent in CB_1_R+/+ groups. Ach-induced endothelium-dependent relaxation was the best in the CB_1_R+/+, LDLR+/+ and CB_1_R+/+, LDLR−/−, CD groups, which was attenuated in mice with the same genotypes under an HFD. Among CB_1_R−/− groups, the CB_1_R−/−, LDLR+/+, CD group showed the best Ach-induced relaxation, which was altered in HFD groups at low concentrations. This improved relaxation effect among CB_1_R-KO animals was even more prominent in the high-fat-diet groups. By analyzing Ach-induced relaxations with the curve-fitting method, we found that EC50 values were improved in the absence of CB_1_Rs in LDL+/+, HFD animals. The significance of CB_1_Rs in vascular damage is further shown by our in vitro inhibitor studies: HFD-induced attenuation of NO-dependent relaxation was partially improved in the absence of CB_1_ receptors, indicated by the attenuated difference in NO-dependent vasorelaxation between CD and HFD groups. Such results are further supported by the eNOS immunohistochemistry, which revealed that the eNOS density of aortas increased in CB_1_R-KO, HFD mice. The inhibition of cyclooxygenase with INDO slightly modulated Ach-induced relaxation, which was significantly decreased at lower concentrations in both the CB_1_R+/+, LDLR−/−, CD and LDLR+/+, HFD groups. There seemed to be a rearrangement of endogenous prostanoid production, too.

### 3.1. Vascular Alterations in Hypercholesterolemic LDLR-KO Mice

Hypercholesterolemia is a definite risk factor for developing CVDs involving AS [6]. In our experiments, an HFD increased the body weight of the mice, which was significant in CB_1_R-WT groups. Heart weight values were only slightly elevated with the HFD (NS). Previous studies have shown that LDLR-KO mice kept on HFDs have developed hypercholesterolemia and AS-developing sclerotic plaques in their aortas [11,13,15]. An HFD is required for the development of atherosclerotic lesions in LDL-receptor-deficient models [38]. In our study, we found an augmented plasma cholesterol level in LDLR-KO mice, which was drastically elevated after 5 months of HFD feeding.

In HFD hypercholesterolemic mice, Ach-induced vasorelaxation was depressed at low concentrations. The inhibition of NOS significantly attenuated Ach-induced vasorelaxation, indicating the crucial role of NO. In HFD-fed animals, the attenuation of Ach-induced relaxation caused by NOS inhibition was weaker compared to CD groups, indicating a lower availability of NO in HFD-fed mice compared to CD groups. This corresponds to the alterations observed in the expression of eNOS. Changes in INDO sensitivity in relation to Ach relaxation point to an altered pattern of endogenous prostaglandin production.

A functional remodeling of the vascular wall occurs in LDLR-KO mice fed with an HFD, even in vascular areas directly not affected by atherosclerotic plaque formation. During the development of AS due to multiple risk factors, including dyslipidemia with high cholesterol levels, a degenerative remodeling of the vessel wall develops, involving vascular inflammation, smooth-muscle cell proliferation, and endothelial dysfunction, which will turn into plaque formation with calcification and necrosis [2,15,39,40,41,42].

Hypercholesterolemia-induced endothelial dysfunction is mediated by cholesterol accumulation and inflammatory responses with altered blood flow profiles in the vessel wall during degenerative remodeling. Endothelial dysfunction is both functional and morphological [1,3,43,44,45]. We have observed functional endothelial dysfunction of the aorta in HFD-fed mice, characterized by depressed NO-dependent relaxation responses. A decreased availability of NO from the endothelium is an important predictor of the development of AS; however, in early stages, this alteration seems to be reversible [46]. Compensatory mechanisms, like perivascular adipose tissue build-up, might postpone the impaired vasodilatory action of endothelial NO [10]. Plaque formation and vulnerability has been assessed using inflammatory biomarkers such as C-reactive protein, tumor necrosis factor-α, interleukins 6, 17A, 18, and 21, and MCP-1, as well as based on CD68- and lipid-positive areas and macrophage accumulation, as these also promote plaque progression [47].

Our myography results have proven the functional vascular remodeling in LDLR-KO animals kept on an HFD, with compromised endothelial dilation due to a reduced NO action. In parallel, the mice’s increased body weight and elevated blood pressure indicate a consequent elevation in the hemodynamic resistance of the circulation.

### 3.2. Vascular Effects of CB_1_ Receptors and Endocannabinoid Signaling, CB_1_R-KO Mice

It has been shown that the ECS contributes to several physiological regulatory mechanisms, including the cardiovascular system. It induces mainly negative inotropic, vasodilator, and hypotensive actions [2,32]. Previous studies have shown that cannabinoid receptor-mediated vascular signaling influences vascular tone by inducing vasodilation either via endothelial cells (endothelium-dependent vasodilation), via vascular smooth-muscle cells, or via perivascular neurons. Such effects have been demonstrated in the aorta and in the coronary and cerebral arteries.

Vascular functions are substantially modified by endothelial factors, such as NO mediating vasodilation, and also by PGs, which can mediate both vasodilation and vasoconstriction [29,48,49,50,51]. Endothelial cells can produce several COX metabolites with diverse actions [51]. Constrictor PGs are released together with the vasodilatory NO, and the effects of this have been found to be altered into vasodilation in exercise-trained animals [29,50]. Such endothelial vasodilation effects are most pronounced in resistance arteries. Endothelial vasodilatory effects are also elicited by endocannabinoids, which are released in the frameworks of signaling mechanisms of several vascular contractile agonists and by exogenously administered CB_1_R agonists [24,32,35,36,52,53,54,55,56,57,58]. Endocannabinoids bind to CB_1_Rs and induce G_i/o_-protein-coupled signaling, resulting in smooth-muscle cell hyperpolarization and vasodilation [24,32,53,55,59]. The endogenous cannabinoid anandamide elicits lasting hypotension and bradycardia through the CB_1_R signaling pathway [55]. Endocannabinoid release during the signaling of calcium-generating GPCR agonists can modulate vasoconstriction via negative-feedback mechanisms, suggesting the existence of a continuous vasodilator tone in the vascular wall through the endocannabinoid pathway [2,24,29,30,34].

In our previous study, we found that female CB_1_R-KO mice exhibited an augmented NO-dependent relaxation in response to Ach and estradiol. We also showed that the presence of vasoregulatory constrictor PGs observed in the aorta of wild-type mice is missing in CB_1_R-KO mice, while NO-dependent vasodilatory effects are augmented in CB_1_R-KO animals; thus, CB_1_R-KO mice have augmented vasodilatory responses [36].

Here, we demonstrate the presence and significant action of these endothelial factors in hypercholesterolemic AS-prone mice.

### 3.3. Role of CB_1_ Receptors in Hypercholesterolemia-Induced Vascular Alterations in CB_1_R–LDLR Double-KO Mice

Previously, a relationship between the ECS and AS development mechanisms was described [37]. We aimed to study the role of the ECS in a hypercholesterolemia-induced AS model by establishing an LDLR–CB_1_R double-KO mouse model, in which we found significant functional vascular remodeling affected by the presence of CB_1_Rs. Our results indicate that an HFD increased systolic and diastolic BP values in LDLR-KO mice, which was attenuated in HFD-fed CB_1_R-KO mice compared to an HFD-fed LDLR-KO–CB_1_R-WT group. Our myography results show that Ach-induced vasodilation is depressed in HFD groups and NO-dependency is significantly attenuated compared to CD groups, whereas the absence of CB_1_Rs (CB_1_R-KO mice) can moderate the deteriorating Ach vasodilatory effect and NO availability. Improved NO availability is also supported by the immunohistochemistry results showing an augmented eNOS expression in CB_1_R−/−, HFD mice compared to CD-fed mice. Thus, we suggest that the absence of CB_1_Rs can restore the deteriorated NO production and can elevate NO levels in HFD-fed animals. These results show that the absence of CB_1_Rs can delay or override the deteriorating functional and structural effects of a serious hypercholesterolemic state in LDLR-KO mice.

It has been shown previously that activation of the ECS has a regulatory role in food intake, appetite regulation, and energy metabolism, while inhibition of ECS signaling may depress food uptake mechanisms and induce weight loss [2,17,60,61,62]. We also measured significantly lower body weight values in our CB_1_R-KO mice, which values were slightly increased under HFD treatment (not significantly in CB_1_R-KO mice). Langbein et al. found that in LDLR-KO mice, exercise training led to a decreased body weight and white adipose tissue mass, but they found that voluntary movement alone was not sufficient to improve vascular function [63]. In our results, improvement in vascular functions when knocking out the CB_1_R gene in LDLR-KO mice did not seem to be connected to reduced plasma cholesterol levels but was instead elicited by direct effects on the vascular wall.

### 3.4. Roles of the Endocannabinoid System and CB_1_ Receptors in Cardiovascular Pathologies, Vascular Remodeling, and Possible Therapeutic Effects

Blood pressure, vasomotor control, cardiac contractility, vascular inflammation, preconditioning, and angiogenesis are affected by the ECS [16]. Components of the ECS can be found in the vast majority of organs in the body, causing wide-ranging effects [64]. Stimulation and overexpression of CB_1_Rs can cause dyslipidemia and obesity, conditions leading to cardiovascular diseases [36]. The ECS might have both beneficial and detrimental effects on different organs depending on the physiological and pathophysiological conditions present. In some cases, the ECS may become over-activated by playing a compensatory protective role in atherosclerosis, inflammatory diseases, cardiovascular diseases, and hypertension, resulting in lowered BP and heart rate, activating cardiovascular CB_1_Rs [16,19,22,26,32,65]. The ECS can have a detrimental effect in pathological states such as hypotension [16], but divergent results have been observed, which might be the result of the triphasic BP effect of cannabinoids [64]. In human experiments, lower doses of THC (30 mg) have been found to elevate BP, while higher amounts (600 mg) lowered it. Also, synthetic CB_1_R agonist WIN55,212-2 elevated BP in normotensive rats, but decreased it in hypertensive conditions [36,66]. In the treatment of cardiovascular diseases such as myocardial infarction, heart failure, atherosclerosis, and cardiometabolic disorders, the use of selective CB_1_R or CB_2_R agonists or antagonists might have a beneficial effect [2,17,19,26,32,52]. Preclinical studies are accelerating the development of more selective drugs with promising results to avoid adverse effects in the abovementioned diseases [67]. Strategies to treat these diseases could include targeting cannabinoid receptors located outside the blood–brain barrier, targeting cannabinoid receptors expressed in a particular tissue, targeting upregulated cannabinoid receptors, selectively targeting cannabinoid CB_2_Rs, or utilizing multi-targeting methods [68]. Drugs targeting CB_1_Rs, CB_2_Rs, TRPV1s, and PPARs have been proven effective in animal models mimicking cardiovascular disorders such as hypertension, atherosclerosis, and myocardial infarction. Agonists are used for the treatment of emesis and stimulation of appetite, as well as in the management of neuropathic pain and symptoms of multiple sclerosis [17,62,68,69]. CB_1_R antagonists have been used to treat obesity and associated metabolic dysregulation [17,70].

Several types of synthetic CB_1_R antagonists have been introduced. In vascular studies, neutral antagonist O2050 and inverse agonists AM251 and SR141715 (rimonabant) have been shown to augment GPCR agonist-induced vasoconstriction, indicating the role of signaling-induced endocannabinoid release in mediating vasodilatory effects on CB_1_ receptors [24,29,30,35]. Also, inhibiting CB_1_Rs has been shown to result in abolished WIN 55,212-2-induced vasodilation [29]. One previous study indicates that the treatment of atherosclerotic LDL-receptor-knockout mice with the selective CB_1_R inhibitor rimonabant can decrease the size of sclerotic plaques in the aorta [71]. Also, in another previous study, Tiyerili et al. found that although the inhibition of CB_1_Rs had no effect on atherosclerotic plaque development, it improved endothelium-dependent vasodilation and decreased oxidative stress in the aorta [72]. They also found that treating cultured vascular smooth-muscle cells with rimonabant reduced the angiotensin-II-mediated production of reactive oxygen species and NADPH oxidase activity [72]. Besides the modulation of CB_1_R signaling, the activation of CB_2_Rs, which primarily localize on immune cells, may ameliorate the extent of atherosclerosis [73]. It has also been revealed that low-dose THC treatment can reduce the progression of AS in mice via CB_2_R-mediated immunomodulatory effects [74]. In clinical studies, rimonabant and taranabant have been used previously for treatment of obesity, but unfortunately, they have produced adverse side effects [2,60,70]. However, new candidate CB_1_R antagonists have appeared with therapeutic potential for the treatment of AS, such as a soybean isoflavone genistein [75], which may also have anti-inflammatory and anticancer therapeutic potentials [76]. Also, focus has been turned to the non-brain-penetrating CB_1_R antagonist AM6545 [77]. Related to the therapeutic potential of CB_1_R inhibition, the development of second- and third-generation compounds is urgent in order to achieve therapeutic effects without side effects [2,60,69].

The ECS also induces vascular remodeling in some cases. In our previous experiments, we found a lowered intima–media ratio in the CB_1_R-KO group together with lower COX-2 and higher eNOS expression in accordance with their functional results [36].

In comparison with these previous findings, the results of the present study also indicate altered functional remodeling in the absence of CB_1_Rs, indicating higher NO availability and eNOS expression and lower blood pressure in HFD cases. We also found that the higher heart weight and the elevated cholesterol levels that developed in HFD-fed AS-prone mice did not change significantly in the absence of CB_1_Rs.

## 4. Materials and Methods

### 4.1. Chemicals

Adrenergic alpha receptor agonist phenylephrine, NO-dependent vasodilator acetylcholine (Ach), nitric oxide synthase (NOS) inhibitor Nω-nitro-L-arginine (LNA), and cyclooxygenase (COX) inhibitor indomethacin (INDO), as well as all other salts and chemicals, were purchased from Merck KGaA (Darmstadt, Germany). Stock solutions of solvents for INDO were prepared in dimethyl sulfoxide (DMSO) and subsequently diluted in Krebs solution on the day of the experiment. A similar dilution of DMSO was used as the “vehicle”. LNA was diluted in Krebs solution with ultrasound dispersion. From the stock solutions, further dilutions were made with Krebs. On the day of the experiment, Krebs solution and high-potassium Krebs solution were prepared using the following components: NaCl, KCl, CaCl_2_·2H_2_O, MgSO_4_·7H_2_O, NaHCO_3_, KH_2_PO_4_, EDTA, and glucose. For a list of the immunohistological reagents and their sources and dilutions, see the corresponding subchapters.

### 4.2. Animals

Homozygous CB_1_R-KO (CB_1_R−/−) and CB_1_R-WT (CB_1_R+/+) mice (Cnr1tm1zim) were obtained in advance by back-crossing chimeric and heterozygous animals to C57BL/6J mice and interbreeding heterozygous animals [78]. They were bred at the HUN-REN Institute of Experimental Medicine’s animal house. LDLR-KO (LDLR−/−) mice were obtained from Jackson Laboratory (B6.129S7-Ldlrtm1Her/J, Jackson Laboratory, Bar Harbor, ME, USA). In order to obtain homozygous double-knockout animals, mice were genetically crossed and bred at the animal facility of the Semmelweis University Basic Medical Science Center. Institutional and national guidelines for animal care and breeding were followed and the study protocol was approved by the Animal Care Committee of the Semmelweis University, Budapest, and by Hungarian authorities (approval no. PE/EA/1428-7/2018 and PE/EA00670-6/2023). Thus, by crossing the LDLR-KO mouse strain with CB_1_R +/− animals, we established a mouse model suitable for studying the involvement of CB_1_Rs in the development of hypercholesterolemia and atherosclerosis.

In our experiments, male mice were used and fed with special diets obtained from Ssniff Spezialdiäten GmbH (Soest, Germany, https://www.ssniff.com, accessed on 1 March 2022), which were given to the animals ad libitum in the form of pellets. Mice were genetically tested and grouped according to their genotype and diet as indicated in Table 1. Groups 1–4 were given a control diet (CD) containing no cholesterol (0%) and decreased levels of crude fat (5.1%), sugar (11.0%), gross energy (18,3 MJ/kg), and metabolizable energy (15.7 MJ/kg), while groups 5–8 were fed with an HFD (Western-type diet) containing elevated levels of crude fat (21.1%), cholesterol (0.21%), sugar (34.3%), gross energy (21.8 MJ/kg), and metabolizable energy (19.1 MJ/kg). Mice started their diet from the age of 1 month and continued for 5 months. Administering a high-fat diet for this length of time has been shown to significantly increase cholesterol levels and plaque formation in LDLR-KO mice [79,80]. The termination age of the mice was uniformly 6 months. The LDLR-KO mouse strain kept on an HFD developed sclerotic plaques in their aortas (Supplementary Figures S2 and S3).

During the experiments, mice were subjected to body and heart weight measurements, cholesterol level determinations, blood pressure measurements, wire myography of their aortic rings, and immunohistochemical staining of the endothelial nitric oxide synthase (eNOS) in their aortic specimens.

### 4.3. Cholesterol Level Determination

At the onset of the experiments, after 2–3 h of fasting, plasma samples were taken from the animals under anesthesia (see details in Section 4.5). Cholesterol levels in the blood plasma of the animals were assessed using the EnzyChrom™ AF Cholesterol Assay Kit (BioAssay Systems, Hayward, CA, USA) according to the manufacturer’s instructions. In summary, cholesterol standards with given concentration values were prepared. Blood plasma samples were subsequently diluted by a factor of one hundred using Assay Buffer (1:100 = plasma/Assay Buffer). Then, 50 µL of a cholesterol standard or plasma sample was aliquoted into corresponding wells of a 96-well plate in duplicates. Equal volumes (50 µL) of the reaction mix, comprising 55 µL Assay Buffer, 1 µL Enzyme Mix, and 1 µL Dye Reagent, were subsequently added to both the cholesterol standards and plasma samples. Incubation lasted for 30 min at room temperature and was followed by optical density (OD) determination at 570 nm.

### 4.4. Blood Pressure Measurement

We measured the blood pressure (BP) of the animals before the experiments with the tail-cuff method under superficial intraperitoneal Euthasol anesthesia, given in a reduced dosage of 35 mg/kg. Blood pressure values were recorded using a CODA tail-cuff blood pressure monitor (Kent Scientific Corporation, Torrington, CT, USA).

### 4.5. Myography

Animals were anesthetized by Euthasol, 55 mg/kg, administered intraperitoneally (additional dosage was administered after BP measurements). The depth of anesthesia was checked by verifying the absence of pain reflexes. The whole circulatory system was perfused with Krebs to remove blood and aortas were dissected. Abdominal aortic segments underwent wire myography measurements, as described before [24,36,79]. Segments were put into cold Krebs solution containing the following (in mmol/L): 119 NaCl, 4.7 KCl, 2.5 CaCl_2_·2H_2_O, 1.17 MgSO_4_·7H_2_O, 20 NaHCO_3_, 1.18 KH_2_PO_4_, 0.027 EDTA, 10.5 glucose. Abdominal aortic rings were cut and mounted onto the wires of the myograph system (610 M Multiwire Myograph System, Danish Myo Technology A/S, Aarhus, Denmark) to record isometric tension. Data were recorded simultaneously on 8 channels via the Powerlab data acquisition system; evaluations were carried out using the LabChart version 8 evaluation software (ADInstruments, Oxford, UK; Ballagi LTD., Budapest, Hungary). The myograph chambers were filled with Krebs solution and maintained at a temperature of 37 °C, aerated with carbogenic gas (95% O_2_ + 5% CO_2_) to keep the pH at 7.4. According to our protocols [24,36], abdominal aortic segments were pre-stretched to 10 mN and were allowed to equilibrate for 30 min. After the equilibration period, a reference contraction (considered 100%) was elicited using hyperkalemic Krebs solution, containing 124 mmol/L potassium. Vasorelaxation effects were tested with the NO-dependent vasodilator acetylcholine (Ach, from 1 nmol/L to 1 µmol/L) after phenylephrine-induced precontraction (10 µmol/L). Selective inhibitors were applied to test the mechanisms of endothelium-mediated relaxation of the aortic rings: NOS was inhibited by LNA and COX was inhibited by INDO, while parallel segments served as controls as they were treated with the vehicle only. Specific inhibitors were applied 20 min prior to the administration of the agonist. Vasodilation responses were determined relative to the precontraction state as percent values, as described previously [24,36,81].

### 4.6. Immunohistochemistry

Paraformaldehyde (PFA)-fixed, paraffin-embedded abdominal aortic sections, 2.5 µm thick, were cut. Sections were immunohistochemically stained against endothelial nitric oxide synthase (eNOS). After deparaffinization, antigen retrieval was performed by heating the slides in citrate buffer (pH = 6). Endogenous peroxidase activity was blocked with 3% H_2_O_2_. To eliminate the nonspecific labeling of the secondary antibody, we used a 2.5% normal horse serum (NHS) blocking solution (Vector Biolabs, Burlingame, CA, USA).

The primary antibody used with overnight application at 4 °C was as follows: eNOS mouse monoclonal antibody 1:50 (Abcam, Cambridge, UK). For secondary labeling, we used horseradish peroxidase (HRP)-linked anti-mouse IgG polyclonal antibodies (Vector Biolabs, Burlingame, CA, USA). Visualization was performed using 3′3-diaminobenzidine (DAB, Vector Biolabs, Burlingame, CA, USA).

Slides were photographed through a Nikon Eclipse Ni-U microscope with a DS-Ri2 camera (Nikon, Minato, Tokyo, Japan). Photos of the slides were taken at 20× magnification. On immunohistochemical slides, the brown positivity and the background staining (DAB and hematoxylin) were separated, and staining intensity was determined based on the noncalibrated optical density. In the case of eNOS, we investigated the staining intensity in the endothelial layer using the FIJI^®^ software (ImageJ 1.54f; Java 1.8.0_322, National Institutes of Health, Bethesda, MA, USA, https://imagej.net/software/fiji/downloads, accessed on 25 May 2024).

### 4.7. Statistical Analyses

In the wire myography experiments, Ach-induced relaxation data were calculated as percent values of the precontraction level. Statistical analysis was performed with a two-way ANOVA and the Bonferroni post hoc test for the analyses of comparisons between the eight groups and a one-way ANOVA and the Kruskal–Wallis test were used to make comparisons at each concentration level. Cholesterol levels were analyzed with a two-way ANOVA and the Holm–Sidak test. Pairwise comparison with the absence and presence of CB_1_ receptors was tested with a one-way ANOVA. Immunohistochemical results were tested with a one-way ANOVA and the Tukey and Holm–Sidak post hoc tests. Emax and EC50 values of Ach-induced dose–response curves were also analyzed with the curve-fitting method (Supplementary Table S1). Values were expressed as the mean ± standard error of the mean (mean ± SEM), and *p* < 0.05 was considered significant. These analyses were performed using the SigmaStat software Version 3.5 (Systat Software Inc., San Jose, CA, USA, accessed by 1 May 2023) with GraphPad PRISM 9.5.0. (San Diego, CA, USA).

## 5. Conclusions

In the present study, we introduce the establishment of an LDLR–CB_1_R double-knockout mouse model, which formed a tool for us to study the involvement of CB_1_Rs in the development of hypercholesterolemia and atherosclerosis. Our results indicate that the functional vascular remodeling of hypercholesterolemic AS-prone LDLR-KO mice fed on an HFD is accompanied by depressed NO-dependent vasodilatation. Our main finding is that this altered functional vascular remodeling effect in HFD-fed mice was partially improved in the absence of CB_1_ receptors. This effect was, at least partially, supported by an augmented eNOS expression. Elevated systolic and diastolic BP values in the LDLR-KO mice were attenuated by knocking out the CB_1_R. HFD-induced vascular remodeling effects can predispose individuals to hypertension and other cardiovascular diseases, which can be partially prevented in the absence of CB_1_Rs. These results suggest novel therapeutic pathways for improving vascular functions in AS and its accompanying comorbidities.

The double-receptor-knockout animal model we have established here, where CB_1_Rs are missing in all tissues throughout the entire life of the animal, gives us a safe foundation for the statement that endocannabinoids and CB_1_Rs are involved in vascular damage induced by hypercholesterolemia. Further studies are needed with additional techniques to reveal more specific molecular mechanisms in vascular and non-vascular tissues, as well as their roles in different stages of atherosclerotic plaque development.

## Figures and Tables

**Table 1:** Grouping of animals by genotype and diet.

Group Number	Genotype	Diet	n
1.	CB_1_R+/+; LDLR+/+	CD	9
2.	CB_1_R−/−; LDLR+/+	CD	6
3.	CB_1_R+/+; LDLR−/−	CD	7
4.	CB_1_R−/−; LDLR−/−	CD	7
5.	CB_1_R+/+; LDLR+/+	HFD	10
6.	CB_1_R−/−; LDLR+/+	HFD	10
7.	CB_1_R+/+; LDLR−/−	HFD	6
8.	CB_1_R−/−; LDLR−/−	HFD	7

Abbreviations: CD, control diet; HFD, high-fat diet; CB_1_R+/+, cannabinoid type 1 receptor wild type; CB_1_R−/−, cannabinoid type 1 receptor knockout; LDLR+/+, low-density lipoprotein receptor wild type; LDLR−/−, low-density lipoprotein receptor knockout; n, number of animals used per group.

## Data Availability

This study’s data are available in the Supplementary Materials.

## Supplementary Materials

The supporting information can be downloaded at https://www.mdpi.com/article/10.3390/ijms25179537/s1.
