# Supplementary material for: Investigating the Role of Cannabinoid Type 1 Receptors in Vascular Function and Remodeling in a Hypercholesterolemic Mouse Model with Low-Density Lipoprotein–Cannabinoid Type 1 Receptor Double Knockout Animals
Source: Int J Mol Sci. 2024 Sep 2;25(17):9537. doi: 10.3390/ijms25179537 (PMC11395437; doi:10.3390/ijms25179537)
Supplement: Supplementary file 1 [file ijms-25-09537-s001.zip › ijms-3133175-supplementary.pdf]

Supplementary Material

Supplementary Figure S1

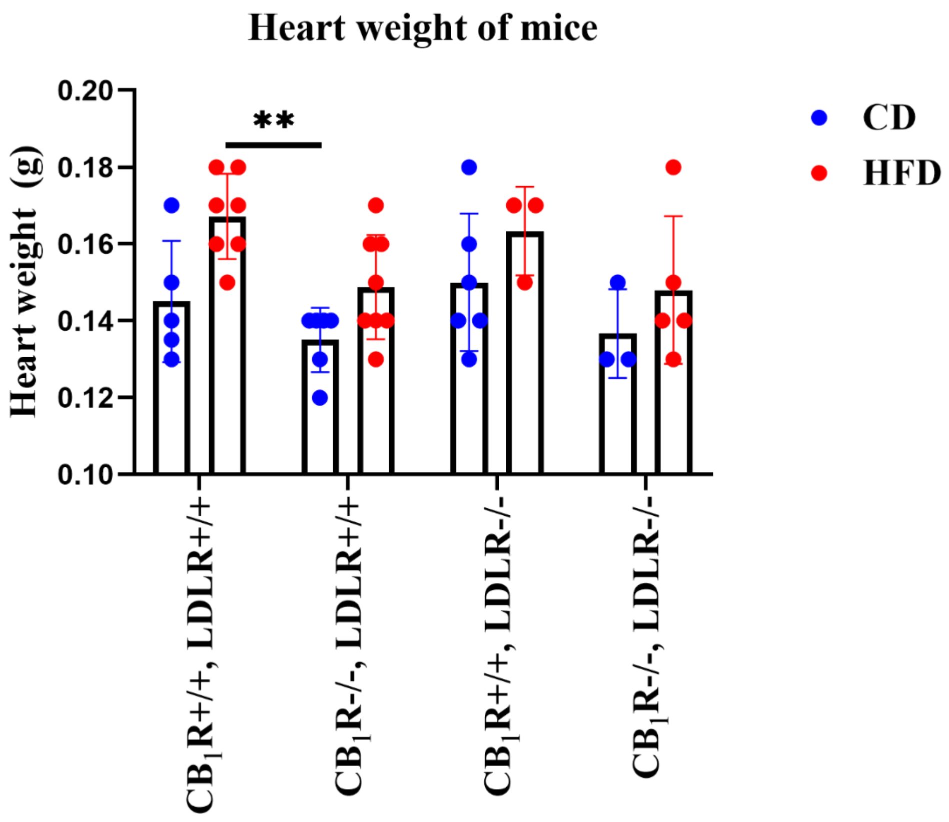

Supplementary Figure S1. Heart weight of control- and high-fat diet-fed CB_1R and LDLR wild type and knockout mice (one-way ANOVA, pairwise comparisons with Bonferroni post-hoc test, **, p<0.007; n=3-8). Heart weight values slightly increased to HFD, which effect was not significant. A significantly elevated heart weight can be seen in CB_1R+/+, LDLR+/+, HFD group compared to CB_1R−/−, LDLR+/+, CD group (p=0.007, abbreviations: CD, control diet; HFD, high-fat diet; CB_1R+/+, CB_1R wild-type; CB_1R−/−, CB_1R knockout mice; LDLR+/+, low density lipoprotein receptor wild type; LDLR−/−, low density lipoprotein receptor knockout mice.

Supplementary Figure S2

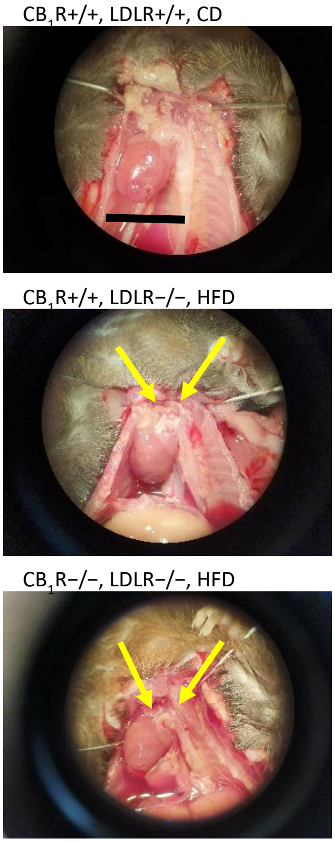

Supplementary Figure S2. Microscopic photos of the chest of mice during preparation to visualize the thoracic aorta. Atherosclerotic plaques developed of LDLR−/− mice kept on 5 months high-fat diet in contrast of control diet animals. Panel (A): CB_1R+/+, LDLR+/+ kept on CD didn't develop plaques in the arch of aorta (control). Panel (B-C): CB_1R+/+, LDLR−/− and CB_1R−/−, LDLR−/− mice kept on HFD has developed sclerotic plaques in the aortic arch shown by arrows. Scale bar shows 1 cm. Abbreviations: CD: control diet, HFD: high-fat diet, CB_1R+/+: cannabinoid type 1 receptor wild type, CB_1R−/−: cannabinoid type 1 receptor knockout, LDLR+/+: LDL receptor wild-type mice, LDLR−/−: LDL receptor knockout mice.

Supplementary Figure S3.

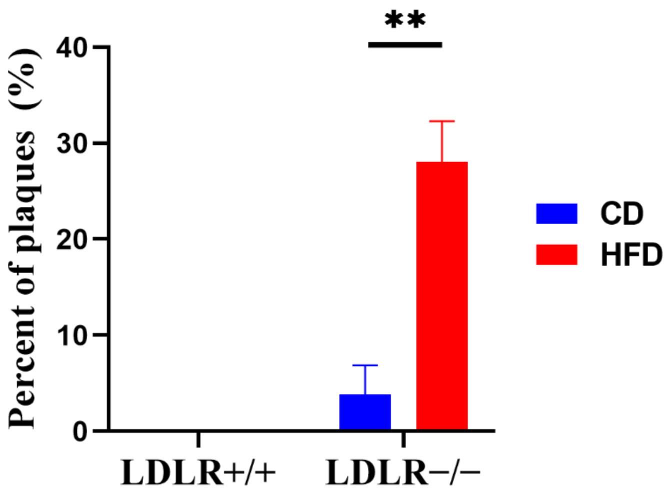

Supplementary Figure S3. Plaque area percentage analyzed with Oil Red plaque staining (Supplementary Method 1) in the thoracic aorta including the arch in LDLR knockout and wild-type mice kept on control diet or high-fat diet. LDLR wild type mice had no plaques developed in the aorta, while LDLR KO mice have developed plaques, which have been significantly greater in high-fat diet animals compared to control diet counterparts (**, p=0.0048, paired t-test). n=3, in all groups. Abbreviations: CD: control diet, HFD: high-fat diet, LDLR+/+: LDL receptor wild-type mice, LDLR−/−: LDL receptor knockout mice, KO: knockout.

Supplementary Table S1

Effective concentration at 50% of maximum response (EC50) in nmol/L and maximum response (Emax) in percent values are shown by analyzing the data of acetylcholine (Ach)-induced dose-response curves in Krebs solution without specific inhibitors with a curve fitting method (related to Figure 4). Statistical differences are shown indicating the groups in comparison. $p<0.05$ was considered significant. There is a significant difference between the CB_1R+/+, LDLR−/−, CD and the CB_1R+/+, LDLR−/−, HFD groups (p=0.043) indicating that HFD treatment ameliorated Ach-induced vasodilation by shifting EC50 value upward. Concerning the effects of CB_1 receptors in Ach-induced vasodilation, there is a significant improvement (decrease in EC50 values) in the CB_1R−/−, LDLR−/−, HFD group compared to the CB_1R+/+, LDLR−/−, HFD animals ($p<0.05$) indicating an improvement in vasodilation in the absence of CB_1Rs. There were no significant differences in Emax values. Statistics were performed with one-way ANOVA and Bonferroni test.

genotype	diet	n value	Emax%	SEM(Emax%)	EC50 nmol/L	SEM(EC50)	Statistics of EC50
CB_1R+/+, LDLR+/+	CD	5	91,5659	4,7777	10,6	2,65	P=0.029 vs. CB_1R+/+, LDLR−/−HFD
CB_1R+/+, LDLR+/+	HFD	10	87,4531	4,645	16,9	4	
CB_1R−/−, LDLR+/+	CD	6	89,5487	5,0845	9,2	2,35	P=0.012 vs. CB_1R+/+, LDLR−/−HFD
CB_1R−/−, LDLR+/+	HFD	8	91,2513	3,8027	14,5	2,4	P=0.04 vs. CB_1R+/+, LDLR−/−HFD
CB_1R+/+, LDLR−/−	CD	7	88,6067	4,8683	13,4	2,93	
CB_1R+/+, LDLR−/−	HFD	5	88,7236	3,6328	26,4	5,3	P=0.043 vs CB_1R+/+, LDLR−/−CD
CB_1R−/−, LDLR−/−	CD	6	87,7655	4,1828	15	3,1	
CB_1R−/−, LDLR−/−	HFD	7	88,0499	4,331	14,5	3,02	P<0.05 vs CB_1R+/+, LDLR−/− HFD

Abbreviations: CB_1R: cannabinoid type 1 receptor, LDLR: low density lipoprotein receptor, −/−: knockout, +/+, wild type, CD: control diet, HFD: high fat diet, n: number of animals per group, Emax%: effective maximum response (%), EC50: effective concentration 50, SEM: standard error of the mean

Supplementary Method

Oil Red staining

Oil Red lipid staining was performed on the upper part of the thoracic aorta including aortic arch to detect sclerotic plaques. During the preparation of the samples, the visible excess fat tissue has been removed from the aortas, then they were kept overnight in 4% paraformaldehyde (PFA), then the vessels were kept in Phosphate-Buffered Saline solution (PBS) at 4 °C until they were stained with Oil Red (Merck KGaA., Darmstadt, Germany). Our protocol for Oil Red lipid stainings was as follows: rinsing samples with PBS, 20 seconds of washing in 60% isopropanol, 10 minutes of Oil Red staining, then once more 20 seconds of washing in 60% isopropanol. At last, samples were rinsed with distilled water, and the stained aortas were kept in PBS. Then the excess fat tissue has been carefully removed from the adventitia, and aortas

were dissected vertically in order to see the inner plaques for further analyses. Photos were taken with a Nikon DS-Ri2 camera attached to a Nikon SMZ25 microscope. Plaque areas of the arch of aorta were selected with free hand in FIJI® software (https://imagej.net/software/fiji/downloads, National Institutes of Health, Bethesda, MA, USA), then the size of the formed plaques (μm^2) was compared to the total area of the utilized vessel.
